# A Blood Screening Test for Dementia with Lewy Bodies for Primary Care

**Published:** 2023-12-04

**Authors:** Melissa Petersen, Tanis J Ferman, Fan Zhang, Otto Pedraza, Zbigniew K Wszolek, Owen A Ross, Tori Como, David Julovich, Leigh A Johnson, James Hall, Neill R Graff-Radford, Sid E O’Bryant

**Affiliations:** 1 Department of Psychology, Institute for Translational Research, University of North Texas Health Science Center, Fort Worth, Texas, USA; 2 Department of Family Medicine, University of North Texas Health Science Center, Fort Worth, Texas, USA; 3 Department of Psychiatry and Psychology, Mayo Clinic, Jacksonville, Florida, USA; 4 Department of Neurology, Mayo Clinic, Jacksonville, Florida, USA

**Keywords:** Dementia with lewy bodies, Parkinson’s disease, Alzheimer’s disease, Proteomics, Blood biomarkers, Biomarker screening, Detection, Diagnostic accuracy

## Abstract

**Introduction:**

We sought to cross-validate our previously published blood test for detecting Dementia with Lewy Bodies (DLB).

**Method:**

Plasma samples were analyzed on 428 individuals (DLB n=89, Parkinson’s Disease (PD) without dementia n=126, Alzheimer’s Disease (AD) n=108, Normal Controls (NC) n=105).

**Results:**

The proteomic profile discriminated DLB and PD from NC with an AUC (Area Under the Curve) of 0.96 with demographics of age, sex and education. The proteomic profile also distinguished DLB from the PD group with an AUC of 0.92 with demographics. It further distinguished DLB, PD and AD from NC with an AUC of 0.95 as well as DLB from AD with an AUC of 0.92 with demographics.

**Discussion:**

This data provides additional evidence of the potential utility of a multi-tiered blood-based proteomic screening method for detecting DLB and distinguishing DLB from NC, PD and AD that can be implemented in primary care settings to aid in the referral process.

## Introduction

With a rapidly aging population globally, primary care providers are faced with the difficult task of detecting dementias in daily clinical practice. Patients with synuclein proteinopathy referred to here as Lewy Body disease (LBD) is the second most common neurodegenerative disease and clinically may present with dementia as Dementia with Lewy Bodies (DLB), Parkinson’s Disease with Dementia (PDD) or without dementia as Parkinson’s Disease (PD). DLB was characterized as a dementia by Kosaka [1] and the first operationalized diagnostic criteria was put forth by McKeith in 1992 [2]. Despite its prevalence, DLB is regularly missed in community clinics [3]. One challenge in screening for such dementia is that the initial symptoms of DLB often differ from those traditionally seen with “dementing illness” and it may be difficult to distinguish DLB from Normal Controls (NC), PD and Alzheimer’s Disease (AD). DLB has been found to be associated with poorer quality of life when compared to those with AD and caregivers of patients diagnosed with DLB often struggle for long periods of time before an accurate diagnosis can be obtained [4]. As with other neurodegenerative diseases, there is a need for screening tools to facilitate diagnosis and help with referrals to specialty clinics for a comprehensive assessment followed by appropriate treatment planning. The goal of this study was to cross-validate our previously generated DLB Blood Test (DBT), which is intended to serve as a screening tool for primary care settings.

It is estimated that DLB accounts for 15–35% of dementia cases [5,6]. However, the prevalence among community care settings is unknown. A recent review found that the average prevalence of DLB in community-based studies was 6.4% [7]. As we have previously published, a blood test that can serve as an initial screening indicating that neurodegenerative disease is highly unlikely (i.e. rule out screening test) would be ideal and offers tremendous utility [8,9]. With a base rate of approximately 6%, a screening test with >90% Negative Predictive Value (NPV) would exclude the vast majority of cases that may not require more invasive and expensive neurodiagnostic procedures. Additionally, such a test may provide an objective method to facilitate the detection of DLB thereby reducing non-essential additional medical procedures and cost. Those who screen positive could then be referred on to a dementia specialty clinic for a more comprehensive examination.

The core clinical features of DLB include parkinsonism, fluctuating cognition, fully formed visual hallucinations and a history of probable Rapid Eye Movement (REM) sleep behavior disorder [10–12]. There is a subset of patients with Lewy body-related pathology who are often not recognized clinically as having DLB [13], in large part because of concomitant AD-related pathology. Further, the more extensive the tau pathology, the harder it is to recognize the DLB phenotype. Multimodality imaging helps to distinguish DLB from AD, but it is an expensive and less viable method for disease detection in community samples [14]. Therefore, a front-line, minimally invasive and cost-effective screening tool for primary care settings would be of tremendous value to the field.

The search for biomarkers with diagnostic and prognostic utility in neurodegenerative diseases has grown exponentially, with the majority of work focusing on neuroimaging [15–18] and Cerebrospinal Fluid (CSF) methodologies [15,17–19]. Some promising evidence suggests that CSF may yield a potential biomarker for α-synuclein but replication with a large sample will be needed [20]. While advanced imaging and CSF methods have tremendous potential as confirmatory diagnostic biomarkers for neurodegenerative diseases, accessibility and cost barriers preclude these from being utilized as a first step in this process [21–23]. A DLB Blood Test would provide the first step in a multi-tiered neurodiagnostic process beginning in primary care settings, which would then allow for more streamlined referrals and also increase appropriate access to more invasive and costlier neurodiagnostic examinations.

There are no currently validated biomarkers for DLB [24]. It has been proposed that biological markers of the clinical conditions associated with DLB should be “cheap, reliable and reproducible and make use of biological samples that are easy to obtain” [22]. Blood-based biomarkers would fulfill these proposed criteria. Additionally, it has been proposed that proteomic biomarker profiling is a promising method for discovering DLB biomarkers [23,24] because a battery of markers covering a range of biological processes may be required to address the needs of such complex disorders [25]. In our recent work, we developed a DLB Blood Test using stored plasma samples from Mayo Jacksonville. In this study, we sought to conduct an initial cross-validation of the DBT in a larger set of samples from Mayo Jacksonville [26].

## Materials and Methods

### Subjects

The study sample included 428 participants (DLB n=89, Parkinson’s Disease (PD) without dementia n=126, Alzheimer’s Disease (AD) n=108, normal controls n=105) seen through the Alzheimer’s Disease Research Center (ADRC) and the Movement Disorders Center at the Mayo Clinic, Florida. All participants underwent a neurologic examination, a Mini-Mental State Examination (MMSE) and clinical diagnosis (AD, DLB, PD) was based on recent criteria [10,27]. Normal controls were recruited through the ADRC and were all determined to have performed within normal cognitive parameters on neuropsychological testing. Informed consent and/or assent was obtained for all participants included in this study.

### Proteomics

Blood samples were collected per the NACC-Alzheimer’s Center guidelines, which also aligns with the recent guidelines published by an international working group [28]. Briefly, non-fasting samples were collected in an Ethylenediamine Tetraacetic Acid (EDTA) tube from participants while seated using a 21 G needle, gently inverted 5–10 times and centrifuged at 2000 × g for 10 min before being aliquoted into cryovial (polypropylene) tubes and stored at −80°C. All processing was completed within a two-hour timeframe. Samples remained in storage until shipped to the University of North Texas Health Science Center Institute for Translational Research (ITR). All samples were assayed in the ITR Biomarker Core. The ITR Biomarker Core utilizes the Hamilton Robotics EasyBlood for blood processing, aliquoting and re-aliquoting. A custom Hamilton Robotics StarPlus system was utilized for preparation of all plates. Proteomic assays were run on a multi-plex biomarker assay platform using Electrochemiluminescence (ECL) per our previously published methods using commercially available kits [8,29]. ECL technology uses labels that emit light when electronically stimulated, which improves the sensitivity of detection of many analytes even at very low concentrations. ECL measures have well established properties of being more sensitive and requiring less volume than conventional ELISAs [30], the gold standard for most assays.

We recently reported the analytic performance of each of these markers for >1,300 samples across multiple cohorts and diagnoses (normal cognition, mild cognitive impairment and AD) [8]. The assays are reliable and our experience with these assays has shown excellent spiked recovery, dilution linearity, coefficients of variation, as well as detection limits. Inter and intra-assay variability has been excellent. Internal QC protocols are implemented in addition to manufacturing protocols including assaying consistent controls across batches and assay of pooled standards across lots. A total of 500 μl of plasma was utilized to assay the following markers (including Coefficient of Variation (CV) and Lowest Level of Detection (LLOD)) with CVs and LLODs calculated from the Meso Scale Discovery (MSD) automated system; fatty acid binding protein (CV=2.2; LLOD=7.4 pg/mL), beta 2 microglobulin (CV=10.1, LLOD=2.9 pg/mL), pancreatic polypeptide (CV=16.2; LLOD=66.7 pg/mL), C-Reactive Protein (CRP) (CV=5.2; LLOD=2.1 pg/mL), Intercellular Adhesion Molecule (ICAM)-1 (CV=6.6; LLOD=1.8 pg/mL), thrombopoeitin (CV=5.9; LLOD=1.5 pg/mL), α2 macroglobulin (CV=3.6; LLOD=339.5 pg/mL), exotaxin 3 (CV=3.3; LLOD=0.7 pg/mL), tumor necrosis factor α (CV=4.2; LLOD=0.05 pg/mL), tenascin C (CV=6.6; LLOD=1.3 pg/mL), Interleukin (IL)-5 (CV=4.4; LLOD=0.05 pg/mL), IL-6 (CV=7.3; LLOD=0.04 pg/mL), IL-7 (CV=1.9; LLOD=0.07 pg/mL), IL-10 (CV=4.6; LLOD=0.02 pg/mL), IL-18 (CV=2.3; LLOD=0.05 pg/mL), I-309 (CV=8.5; LLOD=0.07 pg/mL), Factor VII (CV=8.8; LLOD=2.1 pg/mL), Vascular Cell Adhesion Molecule (VCAM) 1 (CV=5.4; LLOD=5.2 pg/mL), TARC (CV=3.3; LLOD=0.06 pg/mL) and SAA (CV=10.9; LLOD=10.4 pg/mL). The ultrasensitive Simoa platform was used to assay plasma amyloid, tau, Neurofilament Light Chain (NfL) and alpha-synuclein. Intraplate CVs and LLODs were also derived for high and low pooled controls from the Quanterix automated system on the following assays: alpha synuclein (High control CV=0.03, Low control CV=0.03; LLOD=1.0 pg/mL), Amyloid beta (Aβ) 40 (High control CV=0.04, Low control CV= 0.02; LLOD= 0.2 pg/mL), Aβ 42 (High control CV=0.04, Low control CV=0.06; LLOD= 0.05 pg/mL), total tau (High control CV=0.05, Low control CV=0.04; LLOD=0.02 pg/mL) and Nf-L (High control CV=0.07, Low control CV=0.07; LLOD=0.04 pg/mL).

### Statistical analysis

Statistical analyses were conducted using the R (V 3.3.3) statistical software [31], SPSS 24 (IBM) and SAS. Support Vector Machine (SVM) analyses were conducted in a two-step analytic approach to create proteomic profiles specifically for normal control versus Lewy Body Disease (i.e. DLB/PD) and then DLB vs. PD. SVM is based on the concept of decision planes that define decision boundaries and is primarily a classifier method that performs classification tasks by constructing hyperplanes in a multidimensional space that separates cases of different class labels [32] and is amongst the best classification algorithms [33]. Diagnostic accuracy was calculated *via* Receiver Operating Characteristic (ROC) curves. First, SVM analyses were utilized to discriminate normal controls from Lewy Body Disease (i.e. DLB/PD) with resulting diagnostic accuracy statistics generated (Step 1). Next, SVM analysis was restricted only to those with Lewy Body Disease to discriminate DLB from PD (Step 2) with resulting diagnostic accuracy statistics generated. This two-step process was utilized to allow for the overall algorithm to be more robust and avoid multi-level analyses simultaneously, which reduces the risk for error and sample over-identification. In our prior work, we have demonstrated that the overall profile can differ amongst neurodegenerative diseases [29] and therefore, the multi-step process capitalizes on these overall proteomic profile fluctuations. An additional SVM analysis was further conducted to discriminate neurodegenerative diseases (DLB, PD and AD) from normal controls. The SVM analysis was further refined to examine the discriminative performance of the proteomic profile in distinguishing DLB from AD. Finally, in order to provide estimates of the overall utility of the Dementia Blood Test (DBT) in ruling out disease, NPV was calculated using an estimated base rate of 7%.

## Results

Descriptive statistics of the sample are provided in Table 1. When comparing the demographic characteristics, the PD group was found to be significantly younger (p<0.001) compared to the DLB, AD and normal control groups. The AD group had more women (p<0.001), less education (p<0.001) and greater cognitive impairment on the MMSE (p<0.001) when compared to the other groups.

**Figure 1:**
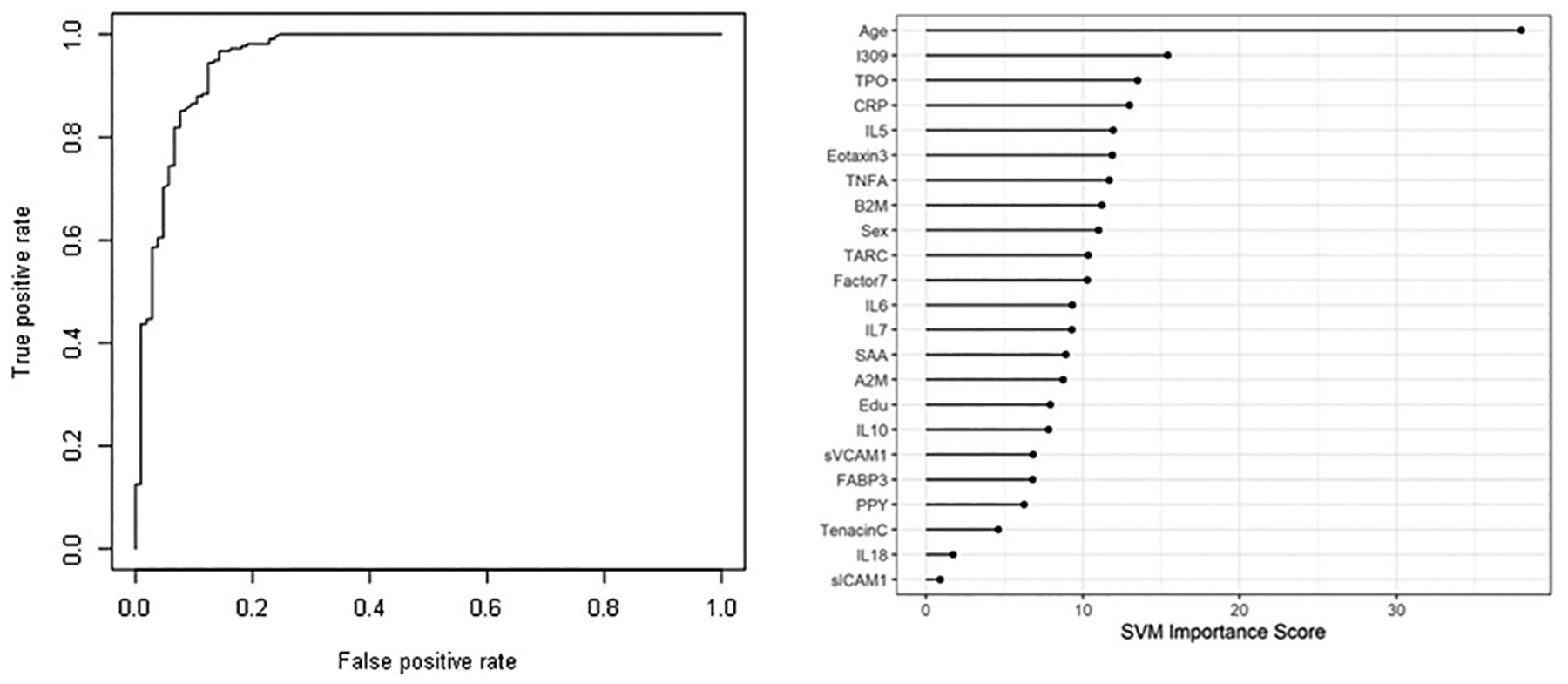
ROC curve and variable importance plot for Step 1–discriminating Lewy Body Disease (Dementia with Lewy Bodies (DLB) and Parkinson’s Disease (PD)) from normal controls with inclusion of age, sex and education.

In Step 1, the DBT was highly accurate in detecting Lewy Body Disease (i.e. DLB and PD) as compared to normal controls. The overall AUC of the proteomic profile was 0.86 with a Sensitivity (SN) of 0.75 and Specificity (SP) of 0.88. As with our prior work, inclusion of demographic variables (age, sex and education) increased the overall accuracy with an AUC of 0.96, SN of 0.88 and SP of 0.94 (p-value for DeLong’s test for ROC curves with demographic variables and without demographic variables is 2.436e-05). Table 2 shows all of the correct and incorrect predictions while the variable importance plot and ROC curve are presented in Figure 1.

**Figure 2:**
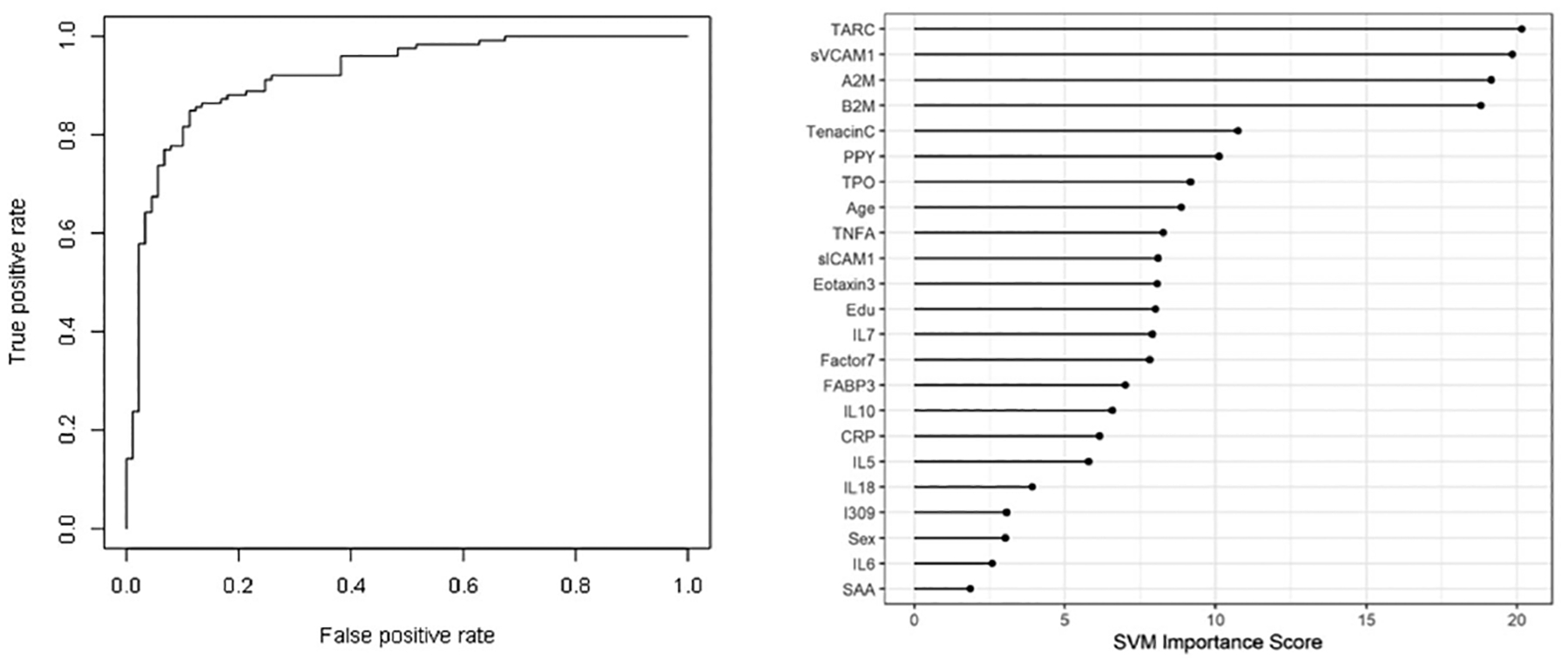
ROC curve and variable importance plot in Step 2-discriminating between Dementia with Lewy Bodies (DLB) and Parkinson’s Disease (PD) with inclusion of age, sex, and education.

In the Step 2, the overall SVM-proteomic profile showed good accuracy at distinguishing DLB from PD. In this model, the AUC was 0.91 with SN of 0.75 and SP of 0.89. Inclusion of demographic variables improved the accuracy to an AUC of 0.92, SN of 0.78 and SP of 0.89 (p-value for DeLong’s test for ROC curves with demographic variables and without demographic variables is 0.1808). Table 3 shows all of the classifications (correct and incorrect) while the variable importance plot and ROC curve are presented in Figure 2.

We further performed 5-fold cross-validation on the two-step analytic approach. We achieved slightly lower performance in the testing set: AUC=0.76 (SN of 0.41; SP of 0.80; PPV12 of 0.22; NPV12 of 0.90) for without demographic variables and AUC=0.84 (SN of 0.59; SP of 0.84; PPV12 of 0.35; NPV12 of 0.94) for with demographic variables.

**Figure 3:**
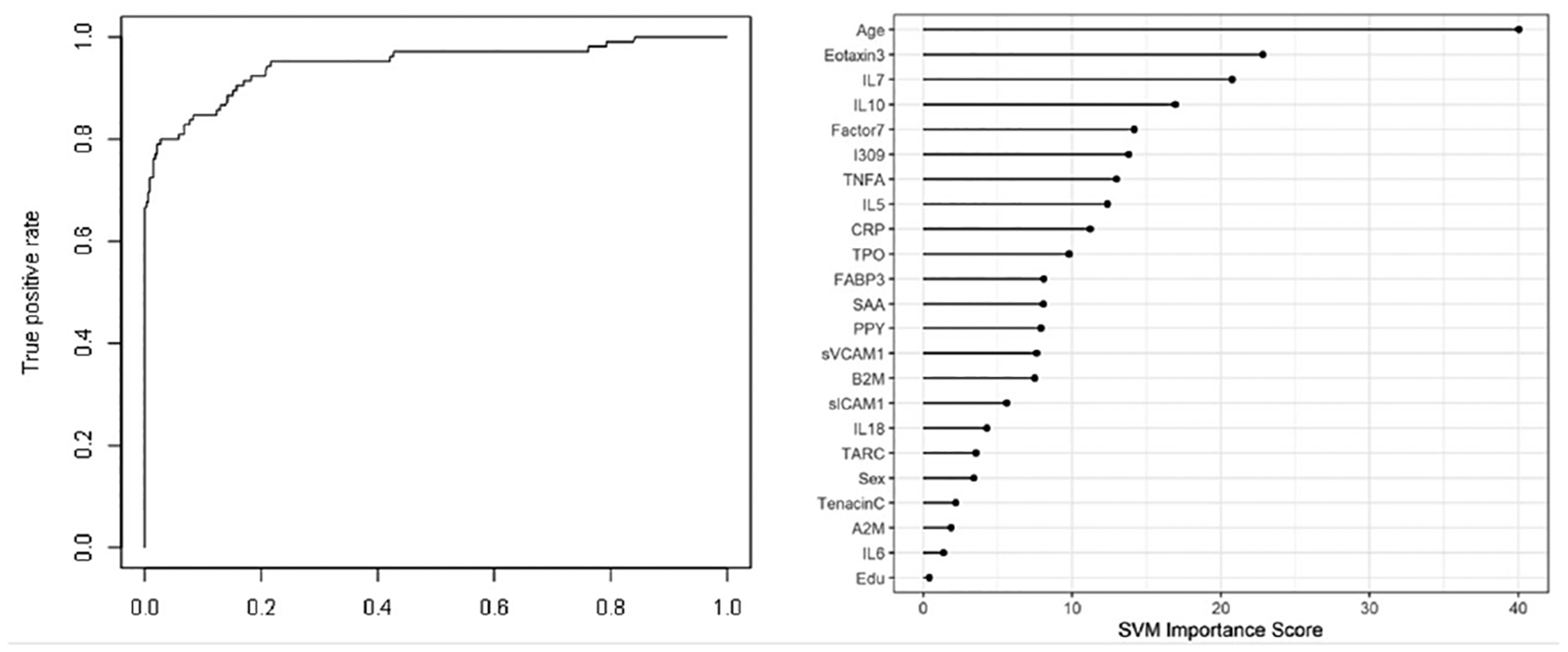
ROC curve and variable importance plot for discriminating Dementia with Lewy Bodies (DLB), Parkinson’s Disease (PD), and Alzheimer’s Disease (AD) from normal controls with inclusion of age, sex, and education.

**Figure 4:**
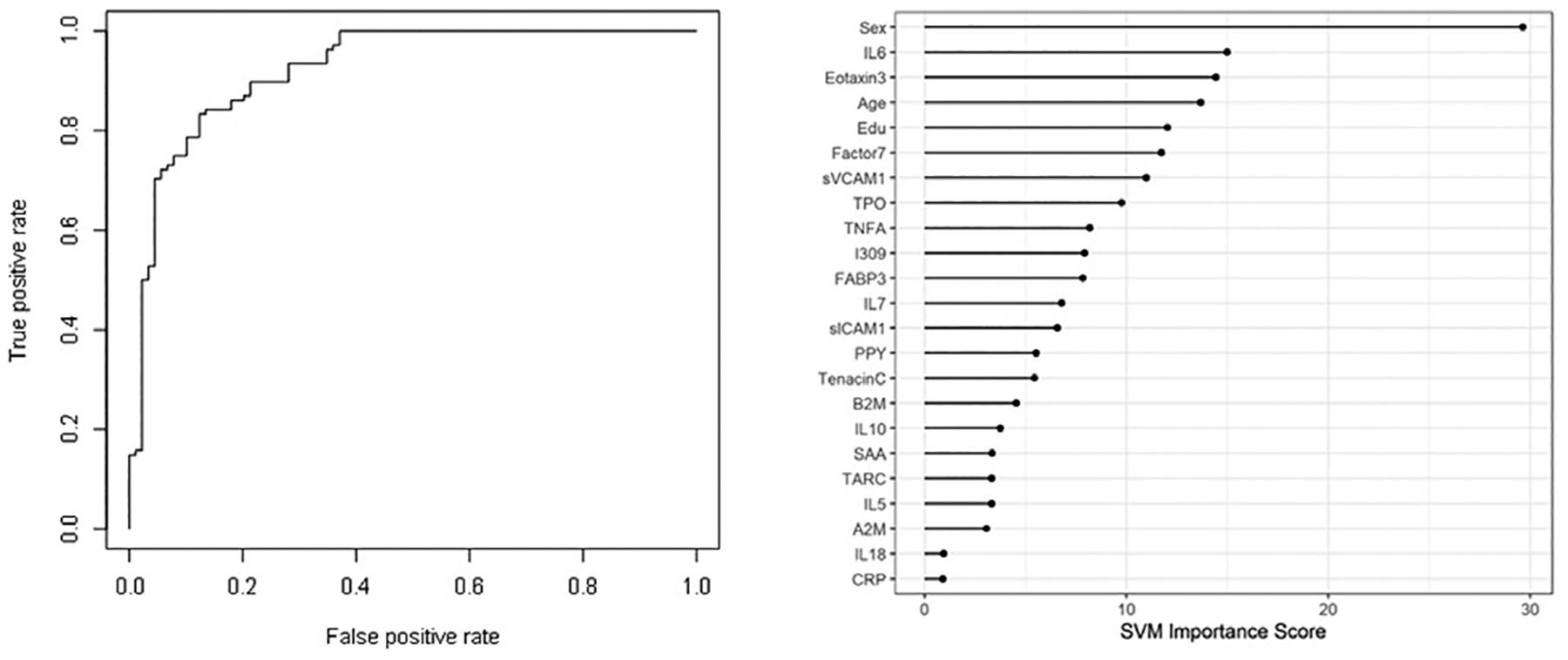
ROC curve and variable importance plot for discriminating Dementia with Lewy Bodies (DLB) from Alzheimer’s Disease (AD) with inclusion of age, sex, and education.

When the SVM-proteomic profile was expanded to differentiate neurodegenerative diseases (DLB, PD and AD) from normal controls, it produced an AUC of 0.95 with SN of 0.99 and SP of 0.70 with the inclusion of demographic variables. Table 3 shows the classification (correct and incorrect) of the proteomic profile while the variable importance plot and ROC curve are presented in Figure 3. When distinguishing DLB from AD, AUC remained elevated at 0.92 with SN of 0.85 and SP of 0.84 with again the inclusion of age, sex and education. Table 4 depicts the classification (correct and incorrect) for distinguishing the two neurodegenerative conditions while the ROC curve and variable importance plots are presented in Figure 4.

Next, the estimated Negative Predictive Value (NPV) was calculated to determine how the DBT would perform as a rule out test in primary care settings. The estimated base rate of 7% was utilized. In order to provide a range, calculations of NPV were also calculated for 5%, 10%, 15% and 20% base rates. The DBT has the potential to be used in primary care settings for yes/no decisions regarding referrals for the presence of DLB. Therefore, the NPV estimates were created for DLB vs normal control. At the estimated base rate of 7%, the NPV of the DBT was 98%. Therefore, if a clinician saw 5,000 patients with complaints per year, n=4,092 patients would immediately be ruled out with only n=88 false negatives. The remaining n=263 would be referred for a dementia specialty clinic examination. NPV estimates for the remaining base rates are as follows: 5% -NPV=99%,10% -NPV=97%, 15%-NPV=95%, 20%-NPV=93%. Therefore, at even an overestimate of base rate, the DBT is highly accurate in ruling out DLB as a possible diagnosis (Table 5).

Follow-up analyses were conducted to examine how inclusion of additional biomarkers related to AD (Aβ40 and Aβ42, total tau), Neurodegeneration (Nf-L) as well as an LBD (α-synuclein) could improve the detection accuracy of the DBT. The overall AUC for Step 1 increased to 0.90 (compared to 0.86) with a SN of 0.74 and SP of 0.93. The inclusion of demographic variables did improve detection accuracy compared to the initial DBT profile, which now reached an AUC of 0.96. When examining Step 2, the AUC increased slightly with the inclusion of added AD and LBD biomarkers to 0.95 (SN of 0.89; SP of 0.87). The inclusion of demographic variables did not improve the detection accuracy greatly with an AUC of 0.96.

When the added AD and LBD biomarkers (along with age, education and sex) were included along with the DBT proteomic profile, it increased the AUC only 1% when applied to the model differentiating neurodegenerative diseases (DLB, PD and AD) from normal controls. Sensitivity and specificity were relatively unchanged (SN of 0.99, SP of 0.69). When distinguishing DLB from AD, the AUC only increased 3% with the added disease-specific biomarkers; sensitivity remained comparable while specificity increased 3%.

## Discussion

The current study demonstrates the potential utility of the DLB Blood Test for use in primary care settings. The context of use would be to specifically serve as the first-step screening tool in a multi-tiered neurodiagnostic process. Given the base rates of disease, the first step needed in primary care is to diminish the chances of those who do not have disease being referred for additional costly and invasive procedures, which is the purpose of our screening tool. As is the case with cancer and other diseases, multi-tiered diagnostic processes are optimal for primary care settings. Rather than a “one size fits all” biomarker, a rapidly scalable, non-invasive and cheap screening test can rule out 90% of patients with concerns who do not need additional testing. This process provides substantial cost savings, reduces burden on specialty clinics, provides medical care providers with a tool for front line screening. For those who screen positive, this may prompt a referral to a dementia specialty clinic for a comprehensive assessment. We propose the DBT as a primary care screening tool and it does not constitute a diagnostic test.

A major advantage to the approach is leveraging of the differing overall profiles, which is captured using advanced SVM-analyses. These results are supported by our prior publications but require additional replication. In our work with the Harvard Biomarkers Study (HBS), we previously demonstrated that our proteomic profile approach could distinguish PD from other neurodegenerative diseases with an overall accuracy of 98% [34]. In our pilot work with the Mayo Clinic Jacksonville Biorepositories, we found that our approach distinguished DLB from PD with an overall accuracy of 92%. We have also shown that the proteomic profile approach can distinguish AD from PD [26].

In our prior work, we have created and validated an AD Blood Test for use in primary care settings across cohorts, species (humans, mice) and tissue (serum, plasma, brain) [8,29,35,36]. Subsequently, we have proposed a multi-tiered neurodiagnostic process for detecting neurodegenerative disease beginning in primary care clinics using blood-based biomarkers [8,37], which is now being prospectively studied in primary care settings (i.e. Alzheimer’s Disease in Primary Care study). The current work is an extension of that work based on the need of primary care settings. The ultimate goal is to provide multiple tools for direct use by PCPs to rule out neurodegenerative diseases. This approach can increase access to more invasive and costlier neurodiagnostic procedures by providing payors with rationale for coverage/reimbursement. Specifically, our blood tests would rule out the large numbers of patients who should not undergo these procedures thereby providing substantial cost savings. The next step is to prospectively apply the DBT to prospectively collected patients seen in community care settings.

There are weaknesses to the current study. First, the patients analyzed in this study were referral samples from dementia and movement specialty clinics rather than a community clinic. Additionally, despite being a sizable proteomic study of DLB, the sample size is still relatively small. Therefore, due to the sample size, the analyses were conducted with internal five-fold cross-validation rather than splitting the cohort into training and test samples. The analyses are also limited in that cross-sectional data was examined and it is possible that longitudinal biomarker changes will refine the overall accuracy. While the addition of novel ultra-sensitive assay of biomarkers related to AD (Aβ40, Aβ42, total tau) neurodegeneration (Nf-L) and LBD (α-synuclein) improved the detection accuracy of the DBT some, it is possible that the inclusion of other biomarkers could aid in the detection of DLB as well as the discrimination of DLB from other neurodegenerative diseases. Prior work has suggested that novel tau-related biomarkers may be of utility, which will be examined once the assays are fully validated and available [38]. Ferman and colleagues recently identified subtypes of LBD associated with α-synuclein and tau distribution, which will be equally important to examine and extend the potential application of the PDBT [39]. It is likely that novel, yet to be studied, biomarkers will aid in the primary care screen as may simple questions for the caregiver (e.g. sleep disturbances), which will be examined in future studies. Taken together, the current findings add substantially to a rapidly growing line of investigation suggesting that blood-based biomarkers can serve in a multi-tiered neurodiagnostic process for detecting a wide range of neurodegenerative diseases, including DLB.

## Conclusion

The continued need for fast, scalable and reliable tools for ruling out diseases has fostered the ever-growing exploration into the utility of blood-based modalities. Prior work has highlighted the feasibility of a blood test to serve as a rule-out mechanism for neurodegenerative diseases. This study validated initial feasibility work and confirmed the application of a DBT for disease detection of DLB. The integration of such a screening test into primary care settings holds considerable application and benefit to patients as well as health care providers particularly as it pertains to access to care.

## Figures and Tables

**Table 1:** Demographic characteristics of cohort.

Category	DLB mean (SD)	PD mean (SD)	AD mean (SD)	Normal control mean (SD)
N	89	126	108	105
Age	73.1(7.7)	69.6(10.5)	74.8(9.3)	85.2(6.5)
Education	15.4(3.4)	16.2(4.0)	13.8(3.0)	14.3(3.2)
Gender (%M)	78.4	71.4	33.3	51.4
MMSE score	21.6(5.9)	28.3(2.1)	17.3(7.5)	28.4(1.9)

**Note:** DLB: Dementia with Lewy Bodies; PD: Parkinson’s Disease; AD: Alzheimer’s Disease: MMSE: Mini Mental State Examination.

**Table 2:** Diagnostic accuracy of blood test in Step 1-discriminating Lewy Body disease (Dementia with Lewy Bodies (DLB) and Parkinson’s Disease (PD)) from normal controls.

Actual		
Predicted	NC	DLB_PD
NC	79%	26
DLB_PD	26%	189
Precision/PPP	75.24%	-
Accuracy	83.75%	-
Sensitivity	75.24%	-
Specificity	87.91%	-
NPP	87.91%	-
AUC	86.04%	-

**Table 3:** Diagnostic accuracy of blood test in Step 2-discriminating between Dementia with Lewy Bodies (DLB) and Parkinson’s Disease (PD).

Actual		
Predicted	DLB	PD
DLB	67	14
PD	22	112
Precision/PPP	82.72%	-
Accuracy	83.26%	-
Sensitivity	75.28%	-
Specificity	88.89%	-
NPP	83.58%	-
AUC	91.01%	-

**Table 4:** Diagnostic accuracy of blood test-discriminating Dementia with Lewy Bodies (DLB), Parkinson’s Disease (PD), and Alzheimer’s Disease (AD) from normal controls with inclusion of age, sex, and education.

Actual		
Predicted	DLB_PD_AD	NC
DLB_PD_AD	321	32
NC	2	73
Precision/PPP	90.93%	-
Accuracy	92.06%	-
Sensitivity	99.38%	-
Specificity	69.52%	-
NPP	97.33%	-
AUC	94.64%	-

**Table 5:** Diagnostic accuracy of blood test-discriminating Dementia with Lewy Bodies (DLB) from Alzheimer’s Disease (AD) with inclusion of age, sex, and education.

Actual		
Predicted	DLB	AD
DLB	76	17
AD	13	91
Precision/PPP	81.72%	-
Accuracy	84.77%	-
Sensitivity	85.39%	-
Specificity	84.26%	-
NPP	87.50%	-
AUC	92.47%	-
